# Quantitative determination of target gene with electrical sensor

**DOI:** 10.1038/srep12539

**Published:** 2015-07-24

**Authors:** Xuzhi Zhang, Qiufen Li, Xianshi Jin, Cheng Jiang, Yong Lu, Roya Tavallaie, J. Justin Gooding

**Affiliations:** 1 School of Chemistry and Australian Centre for NanoMedicine, The University of New South Wales, Sydney, NSW 2052, Australia; 2 Yellow Sea Fisheries Research Institute, Chinese Academy of Fishery Sciences, Key Laboratory of Sustainable Development of Marine Fisheries, Ministry of Agriculture, Qingdao 266071, P.R. China

## Abstract

Integrating loop-mediated isothermal amplification (LAMP) with capacitively coupled contactless conductivity detection (C^4^D), we have developed an electrical sensor for the simultaneous amplification and detection of specific sequence DNA. Using the O26-*wzy* gene as a model, the amount of initial target gene could be determined via the *threshold time* obtained by monitoring the progression of the LAMP reaction in real time. Using the optimal conditions, a detection limit of 12.5 copy/μL can be obtained within 30 min. Monitoring the LAMP reaction by C^4^D has not only all the advantages that existing electrochemical methods have, but also additional attractive features including being completely free of carryover contamination risk, high simplicity and extremely low cost. These benefits all arise from the fact that the electrodes are separated from the reaction solution, that is C^4^D is a contactless method. Hence in proof of principle, the new strategy promises a robust, simple, cost-effective and sensitive method for quantitative determination of a target gene, that is applicable either to specialized labs or at point-of-care.

The determination of target nucleic acids is of great importance for fundamental research and applied technology^1^,^2^. In order to amplify trace amounts of DNA to a detectable level, amplification methods based on various principles have been developed very fast in recent decades^3^. Loop-mediated isothermal amplification (LAMP), which eliminates the rapid thermocycling demand, is highly specific because four primers recognize six separate regions of the target sequence in order for the amplification to proceed^4^,^5^. Moreover, its efficiency is outstanding, as upward of ~10^9^ copies accumulate from less than 10 copies of input template within an hour or two^4^. Hence this gene analysis method is becoming particularly popular and represents a revolution in molecular biology by reducing cost, turnaround time and complexity^4^,^6^.

The efficient determination of a target gene with LAMP relies on the monitoring of amplification reaction. Many methods, such as those based on fluorescence, turbidity, gel electrophoresis, electrochemistry, enzyme-linked immunosorbent assay and lateral flow dipstick, have been developed for monitoring the biochemical reaction^7^. Amongst them, optical and electrochemical methods have been in the forefront because they allow the real time monitoring of the LAMP progression, and provide quantitative reports automatically. However, the optical readout methods require not only highly precise and expensive instruments but also sophisticated numerical algorithms to interpret the data^7^,^8^,^9^. The alternative, electrochemical methods, are faster, lower cost, simpler and can be more readily be miniaturized by eliminating the requirement of photoelectric converter^10^. Unfortunately, for continuously monitoring the progression of the LAMP reaction in real time with contact electrodes, either with voltammetry^11^,^12^,^13^,^14^ or potentiometry^15^,^16^, there is still the issue of low reproducibility due to the fouling and passivation of working electrode, by biological species in the amplification reaction vessel. Furthermore, accompanied with this issue of electrode fouling is the increased risk of carryover contamination. Thus, safer, simpler and more robust methods for monitoring the performance of LAMP reactions are required.

The LAMP reaction can be represented by the following equations:


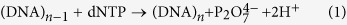



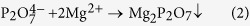



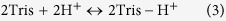


Where dsDNA is synthesized at the expense of consuming primers and dNTPs^8^. An insoluble salt, magnesium pyrophosphate precipitate^17^,^18^,^19^ and protons^17^,^20^ are also produced. The consumption of primers and dNTPs, plus the yield of precipitate, leads to a decrease of the overall ionic strength (as illustrated in Fig 1A), which is what we exploit for monitoring the LAMP reaction via the change in conductivity^21^. Based on this principle, herein an electrical sensor for monitoring the LAMP amplification reaction in real time using a capacitively coupled contactless conductivity detection (C^4^D) was developed (as illustrated in Fig. 1B,C). The new strategy not only has the same advantages that existing electrochemical methods have, but also solves all the challenges existing electrochemical methods face. We anticipate it will enable the creation of a high throughput, portable device for simple, cost-effective and rapid nucleic acid analysis that is suitable both for working in specialized labs and at points of care.

## Results

### Characterization of the electrical sensor

Using 0.1, 0.2, 0.3, 0.4 and 0.5 M KCl as probes, the custom built electrical sensor was characterized, focusing on the sensitivity and stability of the C^4^D (Supplementary Note 1 and Supplementary Fig. 1–3). At room temperature, a sensitivity of 871 mV/M was obtained with the optimal conditions of an excitation amplitude of 16 V and an excitation frequency of 2.0 MHz. Moreover, the temperature inside the sensor can be kept stable at 65 °C. At this temperature the C^4^D also shows high sensitivity and stability.

Using a NMR sample tube as a reaction vessel, a series of LAMP reactions (200 μL per sample) were implemented in the electrical sensor when the temperature was programmed to be 65 °C. We observed that high efficiency and specificity of LAMP reactions could be obtained with this set of primers^22^ using the optimized conditions (Supplementary Note 2 and Supplementary Fig. 4).

### LAMP reaction leading decrease of conductivity response

With 1.25 × 10^4^ copy/μL template DNA or herring sperm DNA, we collected the conductivity responses of the LAMP reaction solutions at room temperature before and after the incubation, respectively. As shown in Fig. 2, the mean output potential value of the negative post-reaction solutions is 1.478 ± 0.001 V. It is not significantly different from the mean value obtained from the same solution prior to performing the amplification reaction. By contrast, the mean value of the positive post-reaction solutions is 1.467 ± 0.002 V, which represents a decrease of ~10 mV compared to the pre-reaction solution, suggesting a significant decrease of conductivity. This result is attributed mainly to the consumption of reactants and the formation of the magnesium pyrophosphate precipitate^21^ (Supplementary Note 3 and Supplementary Fig. 5). In addition, the phosphate backbone of the produced dsDNA in the reaction solution can adsorb positive ions (e.g., [K^+^], [Na^+^], [Mg^2+^]), leading to a decrease of overall ionic mobility^23^. In conclusion, successful amplification leads to a detectable decrease of conductivity response of the LAMP reaction solution; at the endpoint the change of conductivity can be monitored rapidly (<2 s) with the electrical sensor, being free of tube-open operation.

### Real time monitoring the progression of the LAMP reaction

We prepared the positive (containing 1.25 × 10^7^ copy/μL target DNA) and the negative (containing 1.25 × 10^7^ copy/μL herring sperm DNA) LAMP reaction samples at room temperature, followed by loading them into two NMR sample tubes, respectively. Real time collection of the conductivity responses of the reaction solutions in turn was conducted once the reaction tubes were inserted into the electrical sensor, in which the temperature was kept 65 °C. As shown in Fig. 3, for both cases during the first 3 minutes the output potentials increase rapidly because the rise in temperature leads to an increase in ion mobility^24^,^25^. With regards to the negative sample, the output potential comes to a plateau in the following 2 min, and remains stable for all the rest period, indicating no reaction proceeded. In contrast, for the positive sample the output potential begins to drop sharply at the approximately 294th second, indicating the beginning of the detectable decrease of conductivity, which is contributed to the performance of DNA amplification. This time point was defined as *threshold* time (*T*_t_). It is significantly shorter than that observed with either electrochemical methods^11^,^14^,^15^ or optical methods^19^,^26^,^27^, suggesting a faster response. The sharp decline in the conductivity continues for ~110 s, suggesting a rather high efficiency of the biochemical reaction. Thereafter the decrease of output potential slows down to a gentle decline, indicating that the amplification reaction slows considerably, which may be due to the inhibition of polymerase activity by the fall of pH^16^, or the decrease of the concentration of Mg^2+^ 28. Note, the usage of more ThermoPol^®^ reaction buffer than the commended dosage, e.g., ≥1.2×, benefits to obtain stable curves of conductivity responses (see Supplementary Note 4 and Supplementary Fig. 6). In conclusion, the outcomes show that the electrical sensor can not only provide the temperature condition required for the LAMP reaction, but also monitor the progression of the biochemical reaction in real time under the selected conditions.

### Performance of quantifying target gene

Figure 4 shows the typical conductivity responses recorded in real time of LAMP reactions with different concentration of initial template DNA. We observed that the larger the amount of initial template DNA is, the shorter the *T*_t_ is, similar to that appears in real time electrochemical monitoring^11^,^14^,^29^, turbidity monitoring^18^ and fluorescence monitoring^26^,^27^. The insert of Fig. 4 shows the plot of *T*_t_ *versus* log_10_ initial concentration of template DNA. Over the four orders of magnitude concentration range of template DNA investigated, from 1.25 × 10^7^ copy/μL to 1.25 × 10^4^ copy/μL, there is a linear correlation between *T*_t_ versus log concentration. These results show the quantity of the template DNA of an unknown concentration can be determined by comparing the *T*_t_ value with the *T*_t_ values of the template DNA of known concentrations. The velocity of LAMP reaction may depend on the nature of template DNA such as G/C or A/T ratio in amplified region. However, the velocity of LAMP reaction may not affect the quantitative determination by this method since the linearity between *T*_t_ and the initial amount of template DNA is independent of the velocity of LAMP reaction^30^. Using serial dilutions of the O26-*wzy* gene sample, the limit of detection was determined to be 12.5 copy/μL with an incubation time of 30 min, which is lower than that by existing real-time electrochemical method^14^. The sensitivity is 10 times higher than that obtained by conventional PCR method (35 cycles), which is in agreement with previous report^20^.

## Discussion

The portable electrical sensor is composed of two key components. As been characterized, the electrical heater with a programmable thermostat allows us to keep the temperature inside at a desired stable isothermal level; the sensitive C^4^D system allows us to monitor the conductivity response of the solution in the reaction tubes in real time. Hence, simultaneous amplification and detection sequence-specific DNA can be implemented by LAMP. Moreover, it also opens the door to investigate the thermodynamic and kinetic mechanisms of many chemical and biochemical reactions, in which change of ionic activity are involved. Note, the associated electronics could be easily miniaturised to a thumb nail size or less^31^, promising the development of portable and high throughput instrumentations.

In LAMP reaction four or six primers are used to recognize six or eight distinct regions of the target gene sequence, so that the specificity is extremely high^4^,^7^,^8^. Thus, even indirect methods for monitoring the reaction can be employed to perform the determination of target gene^32^. Among the real-time monitoring methods, turbidity shows relatively low sensitivity and slow response^19^. With the help of fluorescence, both the sensitivity and the response speed can be improved significantly at the expense of higher running cost, complex handling procedures^7^,^27^ and non-negligible inhibitory potential from the probes employed^32^. Furthermore, the necessity of optical-electrical signal transferring components increases the complexity in miniaturizing instrumentations and hence manufacturing costs^7^,^33^. These optical-based methods have an outstanding advantage, however, they are free of carryover contamination risk due to the absence of any tube-open operation. By contrast, electrochemical methods, including voltammetry^11^,^12^,^13^,^14^, conductivity^21^ and potentiometry^15^,^16^, have the advantages of not only the inherent miniaturization and portability, but also the independence from sample turbidity, low-cost/low-power requirements and compatibility with microfabrication technology. However, even under optimal conditions there are still several challenges for monitoring the LAMP reaction with electrochemical methods, e.g., inhibition from the indicators and high risk of carryover contamination^7^.

The C^4^D method employed here in the electrical sensor shares all the merits that these existing electrochemical methods have. Moreover, the unique nature, i.e. the separation between the electrode and reaction solution^34^, highlights dramatic advances by solving completely all the problems the electrochemical methods faced, leading to 1) capacity of realizing successive monitoring non-invasively; 2) free of any probes, indicators or labels; 3) complete elimination of carryover contamination risk; 4) extremely simplicity of operation; 5) extremely low cost. Among these advantages listed above, it is worth a special emphasis on the elimination of carryover contamination because the LAMP reaction may lead to incorrect results upon contamination of even a small quantity of amplification product^4^,^8^. In conclusion, the electrical sensor has the advantages that optical and electrochemical methods have, meanwhile eliminates their disadvantages, though at present the sensitivity is a little lower than some other schemes^14^,^32^,^33^ (≤1 copy/μL). The fluctuation of base-line in the present conductivity outputs is another challenge. It is probably be overcome by selecting more suitable reaction vessel. Hence, as a proof of principle, the new strategy promises a superior quantitative determination of target gene, applying either in specialized labs or at the point of care.

## Methods

### The electrical sensor

**Figure 1:**
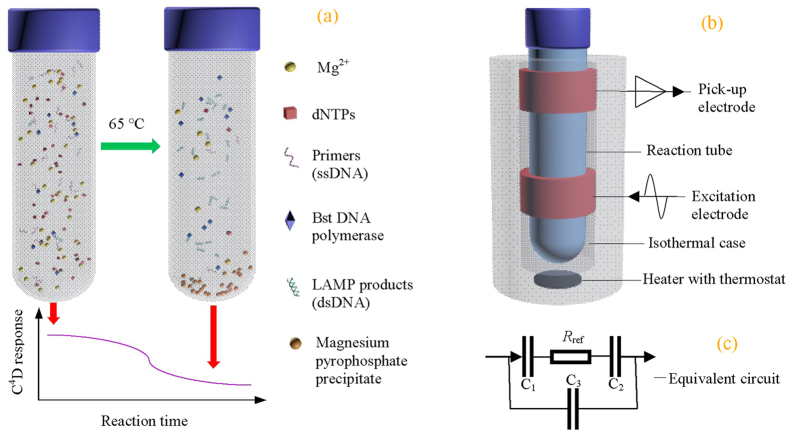
Principle of the electrical sensor for the determination of target DNA by combining LAMP and C^4^D. (**a**) Schematic representation of the LAMP reaction and the consequent conductivity response obtained with C^4^D. (**b**) Structure of the electrical sensor, which includes two electrodes of C^4^D and an electronic heater with programmable thermostat. (**c**) The practical equivalent circuit of the C^4^D detector.

**Figure 2:**
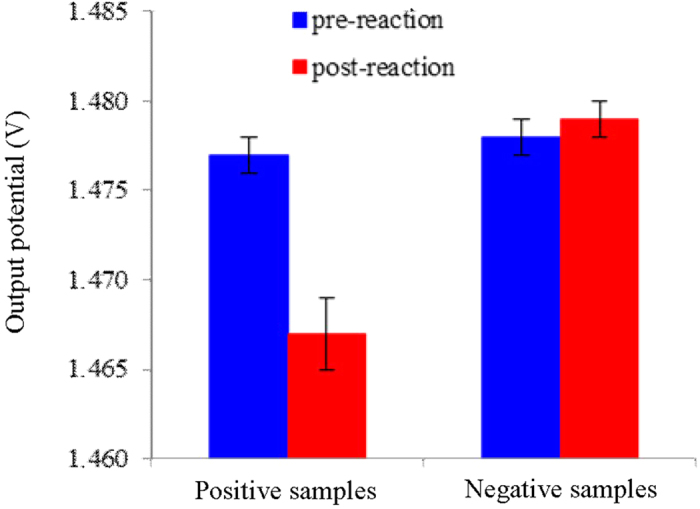
Conductivity responses of pre- and post- LAMP reaction solutions. Five positive samples contained 1.25 × 10^4^ copy/μL template DNA. While the control five negative samples contained the same amount of herring sperm DNA. All the samples were loaded in the same NMR sample tube in turn, and incubated at 65 °C for 12 min, respectively, followed by cooling to room temperature. Conductivity measurements were all carried out with the electrical sensor at 22 °C. Excitation amplitude: 16 V; Excitation frequency: 2.0 MHz.

**Figure 3:**
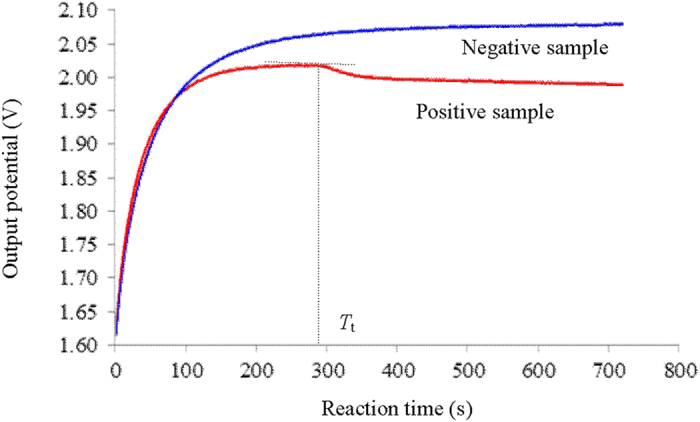
Real time monitoring the conductivity response of the LAMP reactions with the electrical sensor. The positive and the negative LAMP samples contained 1.25 × 10^7^ copy/μL template DNA and 1.25 × 10^7^ copy/μL herring sperm DNA, respectively. The output potential value was collected at 1-second interval. Excitation amplitude: 16 V; Excitation frequency: 2.0 MHz.

**Figure 4:**
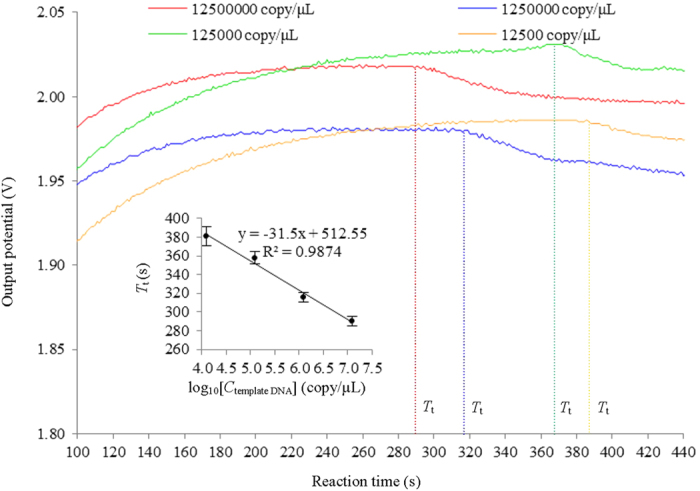
Typical conductivity responses of LAMP reactions with different concentration of initial template DNA. The insert shows a plot of *T*_t_ versus log_10_ initial concentration of template DNA (n = 5). The DNA amplification and monitoring in real time were performed with the same parameters in Fig. 3.

As illustrated in Fig. 1B, the electrical sensor was composed of a C^4^D and an electronic heater with thermostat. The C^4^D included two metal electrodes (an excitation electrode and a pick-up electrode). The practical equivalent circuit is shown in Fig. 1C. The two electrodes, the insulating tube and the electrolyte solution form two coupling capacitances C_1_ and C_2_. And there was also a stray capacitance arising from direct capacitive coupling between the two electrodes through air (C_3_)^34^,^35^. The solution in the reaction tube was equivalent to a resistor *R*. An ER225 C^4^D System (eDAQ Pty Ltd., Australia) was used to provide an AC source (maximum peak to peak amplitude of 40 V) and an AC current pick-up unit. Thus, an alternating current path was formed. The application of an AC voltage on the excitation electrode led to an AC current flowing through the AC path. From the AC current obtained by the AC current pick-up unit, the conductivity detection of the solution in the tube could be implemented. The property of the C^4^D was characterized referring to the method previous reported^24^,^35^,^36^, because the geometry and placement of the sensing electrodes play very important roles in the signal coupling and sensitivity^37^. The electronic heater with a programmable thermostat could provide an isothermal temperature condition (precision: ±0.3 °C), over the range of room temperature −120 °C. Commonly, the temperature could be stable in about 20 min after the appointment.

### LAMP

We retrieved sequences of O-antigen gene clusters of *Escherichia coli* serogroups O26 from GenBank using accession numbers AF529080 (http://www.ncbi.nlm.nih.gov/nuccore/AF529080). Within the cluster, serogroup-specific O26-*wzy* gene was selected as target to design LAMP primers. A dsDNA fragment related to O26-*wzy* gene (190 bp in length), which was inserted in pUC57-Amp (2710 bp in length), was synthesized by GENEWIZ Inc. (USA), and was used as template DNA. Primers were synthesized in Genework Pty Ltd. (Sydney, Australia) with the sequences referred to Wang *et al.*^22^. We listed the detailed data in Supplementary Note 5 and Supplementary Table 1.

The LAMP reaction solution in the NMR sample tube (D = 3.0 mm, Norell, Inc., USA) contained 0.2 μM outer primers, 0.8 μM loop primers, 1.6 μM inner primers, 1.2 mM of each dNTPs, 1.2 × ThermoPol^®^ reaction buffer, 8 μL *Bst* DNA polymerase, 6 mM MgSO_4_ and 8 μL of template DNA. The concentrations of some reactants and supporting species in the LAMP reaction solution, i.e. primers, dNTPs, *Bst* DNA polymerase, ThermoPol^®^ reaction buffer, Mg^2+^ and betaine, which affect the performance of the reaction significantly^4^,^28^, were optimized, referring to the methods reported previously^21^,^29^. We characterized the specificity and efficiency of the reaction with gel electrophoresis^8^,^21^, visual assessment via white precipitate^7^,^20^ and colorimetric detection^8^,^18^,^38^,^39^. A Power PAC300 electrophoresis apparatus and a Gel Doc XR + System (Biorad, USA) were used for agarose gel electrophoresis and detection by employing SYBR GOLD dye (Life Technologies Australia Pty Ltd.). Note, we performed the electrophoresis analysis with 5 times diluted concentration of the post LAMP reaction solution.

### End point detection of the results of LAMP reactions

We prepared a 200 μL LAMP reaction solution at room temperature (22 ± 0.5 °C), followed by loading it into a NMR sample tube. Then the tube was inserted into the electrical sensor, in which room temperature was kept. With an excitation frequency of 2.0 MHz and an excitation amplitude of 16 V, We collected the output potential of its conductivity response. After the reaction solution was incubated at 65 °C for 12 min, it cooled down to room temperature. Then we collected the output potential again with the same parameters.

### Real time monitoring the progression of the LAMP reaction

The preparation and load of the reaction solution was at room temperature. Then we inserted the NMR sample tube loaded with reaction solution into the electrical sensor, in which the temperature was programmed to keep 65 °C. We began to collect the output potential with the ER225 C^4^D System in real time at a speed of 1 point per second from the beginning of the incubation. Unless otherwise stated, an excitation frequency of 2.0 MHz and an excitation amplitude of 16 V were selected; and the record lasted for 12 min. Another sample could be implemented as soon as the former was finished, without the step for renewing working electrodes.

### Performance of quantifying target gene

Serial dilution method was used to study the performance of quantifying O26-*wzy* gene. We prepared a series of LAMP reaction samples, in which contained 1.25 × 10^0^, 1.25 × 10^1^, 1.25 × 10^2^, 1.25 × 10^3^, 1.25 × 10^4^, 1.25 × 10^5^, 1.25 × 10^6^, 1.25 × 10^7^ and 1.25 × 10^8^ copy/μL, respectively. Then we performed the amplification in the electrical sensor by incubating at 65 °C. Meanwhile, we recorded the conductivity responses in real time, respectively. The reaction time, at which the output potential started to drop sharply, was defined as *threshold* time (*T*_t_). Average *T*_t_ from 5 samples for each concentration of template DNA was plotted against log_10_ concentration of template DNA. Error bars represent the variation (RSD) between different samples. Generally, we continued to record the output potential for another 5 min after the appearance of *T*_t_. The PCR control experiments were performed by referencing to Wang *et al.*^22^ with minor modification. In brief, the F3 and B3 were used as the upper- and down-stream primers, respectively. The PCR reaction mixture (25 μL) contained 1 × PCR buffer, 4 mM MgCl_2_, 0.2 mM each dNTP, 0.25 μM each primer, 1.5 U of GoTaq Hot Start Polymerase (Promega, Madison, WI) and 1 μL template DNA. The conditions for the PCR were as follows: denaturation at 94 °C for 20 s, annealing at 60 °C for 30 s, and extension at 72 °C for 50 s in a Mastercycler gradient (Eppendorf, Germany), totally for 35 cycles. The PCR results were evaluated by the electrophoresis with 2% agarose gel.

## Additional Information

**How to cite this article**: Zhang, X. *et al.* Quantitative determination of target gene with electrical sensor. *Sci. Rep.* **5**, 12539; doi: 10.1038/srep12539 (2015).

## Supplementary Material

Supplementary Information
