# Supplementary material for: Quantitative determination of target gene with electrical sensor
Source: Sci Rep. 2015 Jul 24;5:12539. doi: 10.1038/srep12539 (PMC4513347; doi:10.1038/srep12539)
Supplement: Supplementary Information [file srep12539-s1.pdf]

Quantitative determination of target gene with electrical sensor

Xuzhi Zhang[1,2]*, Qiufen Li[2], Xianshi Jin[2], Cheng Jiang[1], Yong Lu[1], Roya Tavallaie[1], J. Justin Gooding[1]

[1] School of Chemistry and Australian Centre for NanoMedicine, The University of New South Wales, Sydney, NSW 2052, Australia

[2] Yellow Sea Fisheries Research Institute, Chinese Academy of Fishery Sciences, Key Laboratory of Sustainable Development of Marine Fisheries, Ministry of Agriculture, Qingdao 266071, P.R. China

Supplementary Note 1

The geometry and placement of the sensing electrodes play a very important role in the signal coupling and sensitivity of the C^4D[1]. So here the properties of the custom built electrical sensor, in particular the nature of C^4D, were characterized, to demonstrate its ability to monitor the progression of the LAMP biochemical reaction.

Because the conductivity response of a solution in the electrical sensor is strongly dependent on excitation frequency and amplitude[2-4], the behaviour of the output potential *versus* the parameters of excitation current was investigated. At room temperature the conductivity responses of 0.1, 0.2, 0.3, 0.4 and 0.5 M KCl solutions were collected in the electrical sensor, respectively. As shown in Supplementary Fig. 1, the output potentials obtained from all solutions rise with the increase of excitation frequency over the range of 0.5-2.0 MHz .From the slope of the lines it can also be seen that the relationship between the excitation frequency and the output potential depends on the ionic strength of the solution tested. Over the range of 0.1-0.4 M, the greater the concentration is, the faster is the increase in the output potential. In previous reports[5,6], it was found that at a certain excitation frequency and a concentration of salt solution, the output potential value would reach a maximum. However, here over the ranges tested, namely, the excitation frequency range of 0.5-2.0 MHz and the concentration range of KCl solution of 0.1-0.5 M, the output potential value does not reach a maximum. So in further experiments, 2.0 MHz, which is the high limit of our C^4D system, was selected as the excitation frequency to obtain the highest sensitivity.

Generally, the higher the excitation amplitudes result in higher output signal strength, better S/N ratio and improved stability[3,4,7,8]. In our experiments, as expected, the output signal strength increases in proportional with the excitation amplitude applied (Supplementary Fig. 2). However, the detection sensitivity does not merely rely on the strength of the output signal. It also depends on the concentration of solution tested. As shown in Supplementary Fig. 2, the output potentials obtained from all solutions rise with the increase of excitation amplitude within 30 V. And the greater the concentration is, the faster is the increase in the output potential, indicating that the relationship between the excitation amplitude and the output potential depends on the ionic strength of the solution tested. When the excitation amplitude is higher, all the output potentials will level off, indicating that excessively high excitation amplitude does no favour for the improvement of analytical capacity. Thus the excitation amplitude of 16 V was selected for the other measurements.

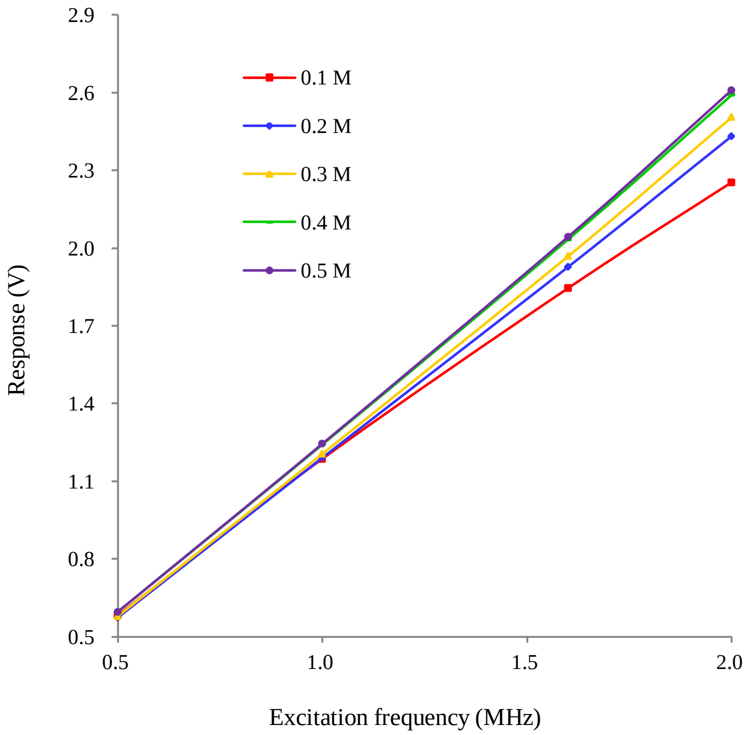

Supplementary Figure 1 Relationships between conductivity responses of 0.1 M, 0.2 M, 0.3 M, 0.4 M and 0.5 M KCl solutions and the excitation frequency. Excitation amplitude: 16 V. The signals were collected at room temperature.

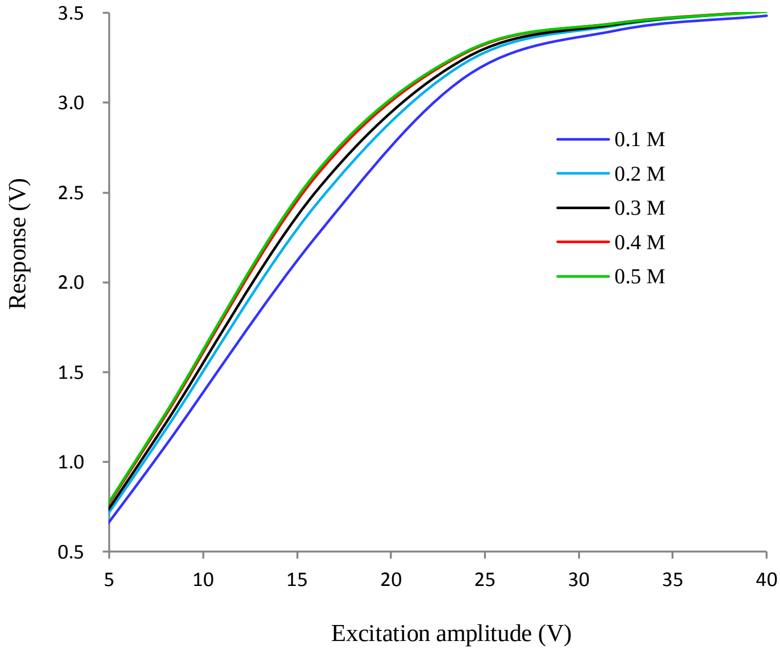

Supplementary Figure 2 Relationships between conductivity response of 0.1 M, 0.2 M, 0.3 M, 0.4 M and 0.5 M KCl solutions and the excitation voltages with excitation frequency of 2.0 MHz. The output potential signals were collected at room temperature.

Conductivity measurements have a high intrinsic temperature coefficient. In practical terms the behaviour of a C^4D system relates closely to the working temperature[4,8,9]. Thus the property of the electrical sensor was investigated as an in-process measurement by changing the working temperature. We began to record the conductivity responses successively as soon as these NMR sample tubes, in which 200 μL of 0.1, 0.2, 0.3, 0.4 and 0.5 M KCl solutions were loaded, respectively, were inserted into the electrical sensor. With an excitation frequency of 2.0 MHz and amplitude of 16 V, five curves were obtained when the solution temperature rose from 22 ℃ to 65 ℃ (as shown in Supplementary Fig. 3). The correlation between the resistance and the temperature[10]:

$$R_T = R_0 (1 + \alpha (T - T_0)) \qquad (1)$$

Where R_T and R_0 represent the resistances of the sensor at temperatures T and T_0 (reference temperature), respectively, and α is the temperature coefficient of resistance. KCl solutions were prepared, and loaded into NMR sample tubes at room temperature. The output potential signals were recorded once per second in real time as soon as the tubes were inserted into the electrical sensor, in which the temperature was 65 ℃. During the period (~60 s) just after the beginning of incubation, the output potentials increase rapidly due to the rapid rise of temperature, which causes the ion mobility to increase[4,9]. When the temperature of the salt solutions reached 65 ℃, the values of output potential all reach plateaus (Supplementary Fig. 3A), suggesting the electrical sensor exhibits a satisfactory stability over the working temperature over range of 22-65 ℃. It is worth noting that the linearity obtained at 65 ℃ is better than that obtained at 22 ℃, though the sensitivity is lower (Supplementary Fig. 3B). Further the conductivity response of 0.1 M KCl solution was recorded successively in the electrical sensor for 2 h at 65 ℃ with the same parameters. During the long period of exposure, no significant deviation of output potential was observed (data not shown), indicating that the C^4D system is stable at high temperature and the deviation of temperature can be negligible, similar to the high temperature C^4D built purposely by Collins et al[9]. The tube loaded with 200 μL0.1 M KCl solution was inserted into the electrical sensor for 5 times. When the temperature reached 65 ℃, the conductivity response was collected, respectively. Results show that the relative standard deviation (RSD) of the output potential values is 1.1%, suggesting a good reproducibility.

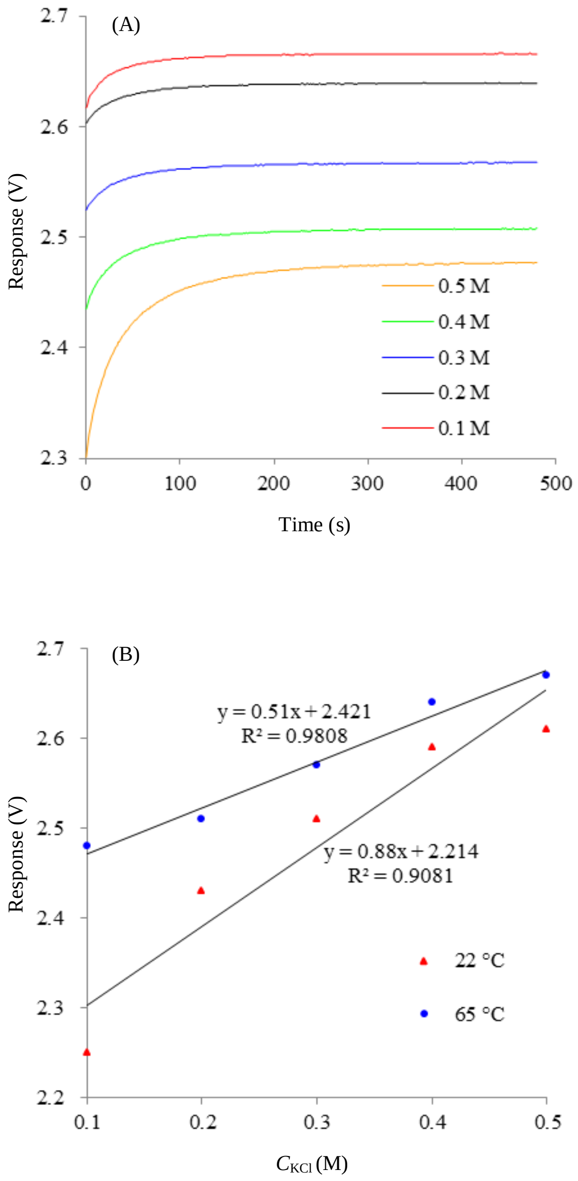

Supplementary Figure 3 (A) Conductivity responses of 0.1, 0.2, 0.3, 0.4 and 0.5 M KCl solutions collected with the electrical sensor. KCl solutions were prepared, and loaded into NMR sample tubes at room temperature. The output potential signals were recorded once per second in real time as soon as the tubes were inserted into the electrical sensor, in which the temperature was kept 65 ℃. (B) The plot of the concentration of KCl solutions *versus* output potentials obtained at 22 ℃ and 65 ℃, respectively. Excitation frequency: 2.0 MHz; Excitation amplitude: 16 V.

Supplementary Note 2

With respect to detecting the result of the LAMP reaction, or monitor the progression of LAMP reaction by measuring conductivity, the ionic strength of the initial reaction solution plays a key role[1]. To maintain a balance between the amplification efficiency and the detection sensitivity by C^4D measurement, we identified an optimum recipe as following: 0.2 μM outer primers, 0.8 μM loop primers, 1.6 μM inner primers, 1.2 mM of each dNTPs, 1.2× ThermoPol® reaction buffer, 0.32 U/μL *Bst* DNA polymerase and 6 mM Mg^{2+}. According to the optimum recipe, a 150 μL LAMP reaction solution was prepared, containing 1.25×10^5copy/μL template DNA and 0.12 mM hydroxynaphthol blue. The solution was loaded into 0.1 mL Rotor-Gene® style tubes by 25 μL per tube. The six tubes were immersed into different glass beakers, in which loaded hot water at 61, 62, 63, 64, 65, or 66 ℃, respectively. It was found that 63 ℃was the lowest temperature for the effective performance of DNA amplification. Whilst, it was also found that the shortest time for the violet reaction solution to become sky blue is obtained by incubation the reaction solution at 65 ℃, suggesting that under this temperature condition the amplification reacts fastest. Thus in the other experiments this incubation temperature was selected as proposed previously[2-5].

With 1.25×10^5copy/μL initial template DNA or the same quantity of herring sperm DNA, a series of LAMP reactions was performed using the parameters selected above. After the incubation of 12 min the reactions were all terminated by heating the mixtures at 80 ℃ for 2 min[2,5]. Then the results of the reactions were characterized with both gel electrophoresis and visual assessments. As shown in Supplementary Fig. 4A, in the four lanes with the template DNA, there are many bands of different sizes in a reproducible ladder-like pattern due to a series of reaction products of different length[2,3]. Meanwhile in the lanes for the negative and control samples (using herring sperm DNA or water take the place of positive template DNA), there are no visible ladder-like patterns. Note, it's hard to recognize the amount of initial template DNA based on the fluorescence intensity, which depends on the amount of product DNA. Supplementary Fig. 4B shows the results of LAMP reactions after keeping the tubes at room temperature for 2 h. With regard to the samples containing template DNA, at the bottom of the tubes there is visible white precipitate, which is insoluble magnesium pyrophosphate produced during the reaction[6-8]. Besides, the white precipitate is stable for more than 3 weeks at room temperature. Meanwhile in the control and negative samples there is no visible precipitate with the same performance. Adding Mn^{2+} and calcein, a fluorescent metal indicator, to the reaction solution allows a visualization of substantial alteration of the fluorescence during the one-step amplification reaction[3,7]. As is evident, only the positive samples become bright green after the incubation (Supplementary Fig. 4C). Similarly, the other kind of fluorescent dye, hydroxynaphthol blue, which is sensitive to the concentration of Mg^{2+}, was also employed to character the result of LAMP with one-step and two-step methods. As expected, the colour of the reaction solution changes from violet to sky blue only after a successful LAMP with positive template DNA[9-11] (Supplementary Fig. 4D). In conclusion, the results of the characterization demonstrate that the LAMP reaction is effective under the selected conditions, as well as keeping the merit of specificity[1].

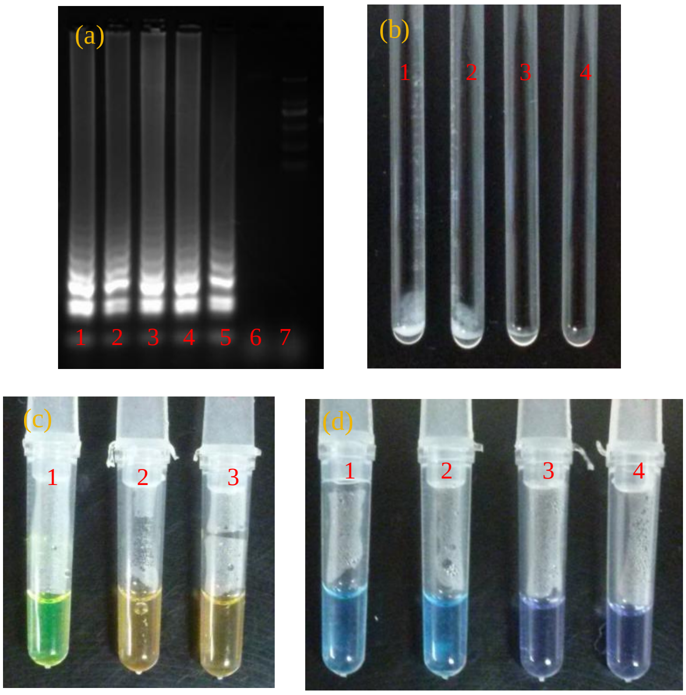

Supplementary Figure 4 Analysis of the results of LAMP reaction. (A) Gel electrophoresis: Lane 1, with 1.25×10^{8} copy/μL target DNA; Lane 2, with 1.25×10^{7}copy/μL target DNA; Lane 3, with 1.25×10^{6} copy/μL target DNA; Lane 4, with 1.25×10^{5}copy/μL target DNA; Lane 5, with 1.25×10^{4}copy/μL target DNA; Lane 6, with 1.25×10^{5}copy/μL herring sperm DNA; Lane 7, without template DNA. Amplicons were analyzed by 2% (w/v) agarose gel electrophoresis at 80 V. (B) Visual assessment via white precipitate: Sample 1, with 1.25×10^{6}copy/μL target DNA; Sample 2, with 1.25×10^{5}copy/μL target DNA; Sample 3, with 1.25×10^{5}copy/μL herring sperm DNA; Sample 4, without template DNA. (C) Colorimetric detection by employing calcein indicator under daylight: Sample 1, with 1.25×10^{5}copy/μL target DNA; Sample 2, with 1.25×10^{5}copy/μL herring sperm DNA; Sample 3, without template DNA. 0.4 mM Mn^{2+} ion and 20 μM calcein were added into the LAMP reaction mixture solution before the incubation. (D) Colorimetric detection by employing hydroxynaphthol blue indicator under daylight: Sample 1, with 1.25×10^{5} copy/μL target DNA and 0.12 mM hydroxynaphthol blue added into the reaction solution before the incubation; Sample 2, with 1.25×10^{5}copy/μL target DNA and 0.12 mM hydroxynaphthol blue added into the reaction solution after the incubation; Sample 3, with 1.25×10^{5}copy/μL herring sperm DNA and 0.12 mM hydroxynaphthol blue added into the reaction solution before the incubation; Sample 4, without template DNA and 0.12 mM hydroxynaphthol blue added into the reaction solution before the incubation. LAMP recipe: 0.2 μM outer primers, 0.8 μM loop primers, 1.6 μM inner primers, 1.2 mM of each dNTPs, 1.2× ThermoPol® reaction buffer, 0.32U/μL *Bst* DNA polymerase and 6 mM Mg^{2+}. Incubation: 12 min at 65 ℃.

Supplementary Note 3

The conductivity properties of some species, which are involved in the LAMP reaction, were investigated with the electrical sensor at both room temperature and 65 ℃. As shown in Supplementary Fig. 5, for all the four kinds of species, i.e. $MgSO_4$, dNTPs, ssDNA and dsDNA, the output potentials increase with the concentration of their solution over the ranges measured. It is worthy of note that for $MgSO_4$ and dNTPs the sensitivity of detection is higher at 65 ℃. Namely, the change of their concentration leads to a more significant change in conductivity at the temperature for the practical LAMP reaction. With regard to ssDNA and dsDNA, it can be seen from the slopes that the conductivity response of the former is far more sensitive than that of the latter. These phenomena are of great benefit for monitoring the result of LAMP reaction, at end-point or in real time.

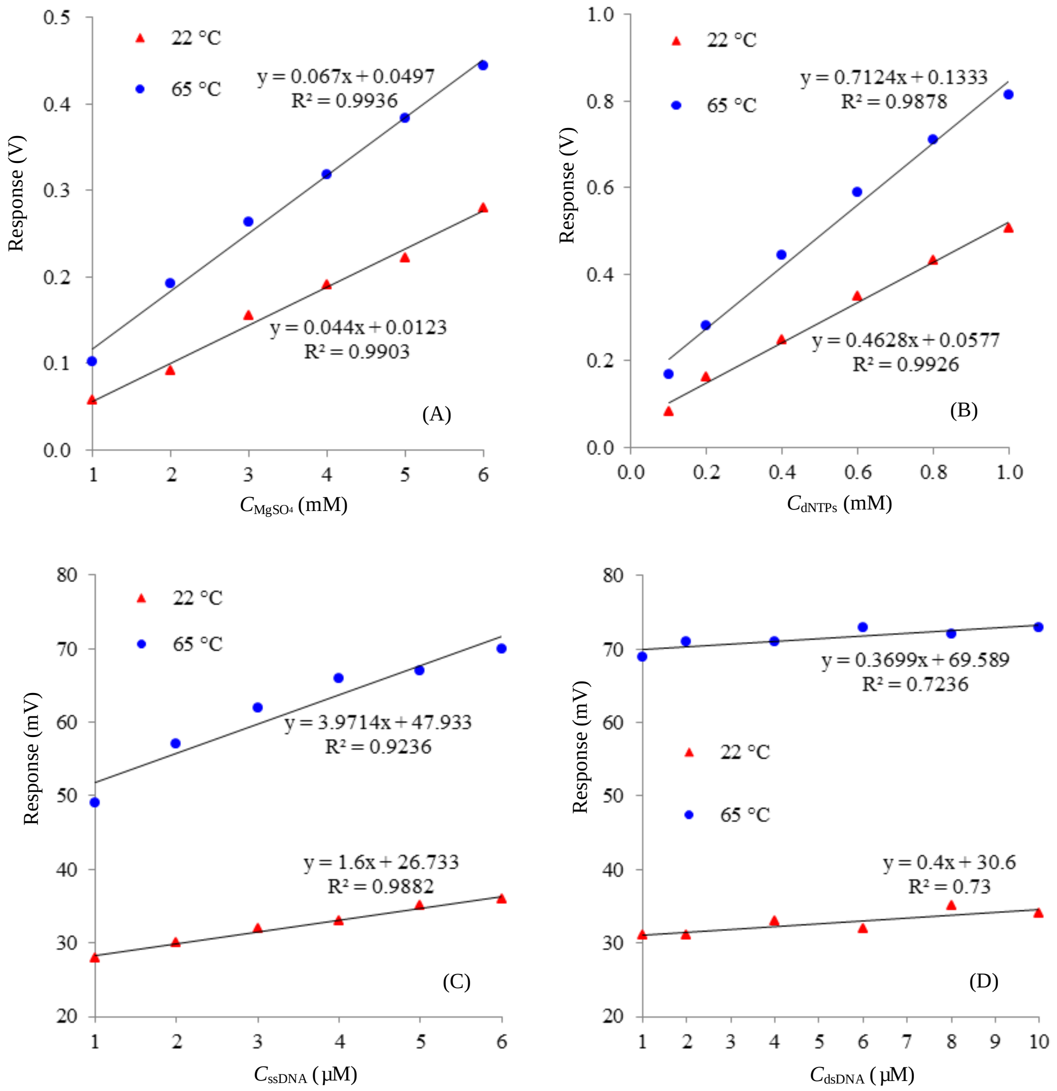

Supplementary Figure 5 Relationships between the concentrations of (A) $MgSO_4$, (B) dNTPs, (C) ssDNA and (D) dsDNA solutions and the conductivity responses at 22 ℃ and 65 ℃. Excitation frequency: 2.0 MHz; Excitation amplitude: 16 V.

Supplementary Note 4

The ThermoPol® reaction buffer plays an important role on the conductivity response. Aiming to obtain a recipe of LAMP reaction solution with low initial ionic strength, 0.8×ThermoPol® reaction buffer was used for the LAMP reaction referred to Veigas*et al*[14]. However, as shown in Supplementary Fig.6, following the drop in potential after the initial stage of heating and the onset of the amplification reaction the output potential shows significant fluctuations. To ascertain the reason for these fluctuations more LAMP reactions experiments were conducted with 1.4×,1.2×,1.0×, 0.8× and 0.6×ThermoPol® reaction buffer, respectively. After the incubation for 12 min the positive results were validated by employing hydroxynaphthol blue. It was found that the pH of all the post-reaction solutions decrease invisibly after the amplification reaction. This is attributed to the hydrogen ions released into the solution during the amplification reaction[16-18]. With regard to those samples with higher ratios of ThermoPol® reaction buffer (1.4× and1.2×), the post-reaction solutions are pH 8.01±0.07 (n=5, measured with Mettler Toledo™ Seven Compact pH/Ion Bench top Meter). By contrast, the post-reaction solutions are pH 7.52±0.13 (n=5) with a lower ratio of ThermoPol® reaction buffer (0.8×). Thus the hypothesis proposed here is in the early stage the *Bst* DNA polymerase incorporates deoxyribonucleoside monophosphates from corresponding dNTPs, releasing pyrophosphate ions and protons. Those pyrophosphate ions combine with magnesium ions immediately because the latter is abundant. The protons combine with unprotonated Tris molecules. Plus during the initial consumption of dNTPs in the reaction, the overall ionic strength of the mixture solution decreases rapidly, leading to a sharp decline of conductivity. Of course, to some extent the increase in dsDNA produced can offset this decline in conductivity. However it can be seen from Supplementary Fig 5C and D that the extent of offset is far weaker than that contribution by the consumption of primers. Thus, the plot of output potential values and the reaction time is a stable inverse ratio curve. With the proceeding of the reaction, at a point the buffer capacity becomes overloaded. At this stage, the generation of ions provide a contribution to increase the overall conductivity of the mixture. While the combination of pyrophosphate ion and magnesium ion and the consumption of dNTPs still lead to the decrease. They are not in balance at different time points, causing the fluctuations of the output potential. In conclusion, 1.2×ThermoPol® reaction buffer can maintain a consistent pH in order to maximize polymerase activity[22], as well as not contribute significantly to the ionic strength to the initial reaction solution (compared to higher dosage, e.g., 1.4×). Hence it was selected for all the other LAMP experiments.

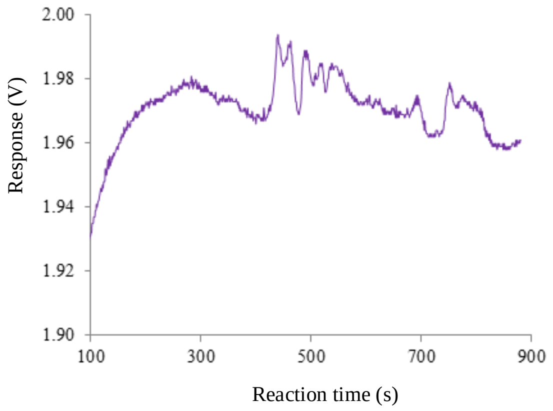

Supplementary Figure 6 Real time monitoring the conductivity response of the LAMP reaction with the electrical sensor. The LAMP reaction contained 0.8× ThermoPol® reaction buffer. The output potential value was collected at 1s interval. Excitation amplitude: 16 V; Excitation frequency: 2.0 MHz.

Supplementary Note 5

Sequences of O-antigen gene clusters of *Escherichia coli* serogroups O26 was retrieved from GenBank using accession numbers AF529080 (http://www.ncbi.nlm.nih.gov/nuccore/AF529080). Within the cluster, serogroup-specific O26-*wzy* gene was selected as target to design LAMP primers. The template DNA related to O26-*wzy*gene (190 bp in length) had the sequence as following (5'–3') GAA TCA AGA CTA TGA AGC GTA TGT TGA TAT ATT TAA TGT CAA TGA ACT TTA TGC CGA GAT TGG TTA TCG CTG GTT AAT TTA TGG TGT TAA GTA TTT AGG CGG TAC CCA TGA AGT CAT AAT TGG CTT GCT GGG TTT ATT CCT TGG GAC CAC ATT CCT ACG ATT AAT ACA ATA CAG TAA GTA TAC AGC ATT T. Primers were synthesized in Genework Pty Ltd. (Sydney, Australia) with the sequences according to Wang *et al*[15] (Table 1). The other ssDNA (80 bp in length) with a sequence of 5'-GAA TCA AGA CTA TGA AGC GTA TGT TGA TAT ATT TAA TGT CAA TGA ACT TTA TGC CGA GAT TGG TTA TCG CTG GTT AAT TT-3' and its exactly complementary target single chain in the same length were also synthesized by Genework Pty Ltd. And they were mixed in water and incubated for 2 h at 42 ℃[23] with gentle stirring to obtain a definite dsDNA.

Table 1 Primers used for the LAMP targeting the O26-*wzy* gene.

Primer description	Primer name	Length (bp)	Sequence (5'–3')
Forward outer	F3	21	GACTATGAAGCGTATGTTGAT
Backward outer	B3	22	TCCTGATTTGAACAATGTCAAT
Forward inner	FIP	48	ACCGCCTAAATACTTAACACCAT AATTAATGTCAATGAACTTTATGCC
Backward inner	BIP	39	TTCCTTGGGACCACATTCCT ACATGTAAAGCAGCAAACC
Loop forward	Loop F	19	ACCAGCGATAACCAATCTC
Loop backward	Loop B	25	TACAATACAGTAAGTATACAGCATT

Supplementary References

1. Mahabadi, K.A. *et al.* Capacitively coupled contactless conductivity detection with dualtop–bottom cell configuration for microchip electrophoresis. *Electrophoresis* **31**, 1063-1070 (2010).
2. Brito-Neto, J.G.A., da Silva, J.A.F., Blanes, L. & do Lago, C.L. Understanding capacitively coupled contactless conductivity detection in capillary and microchip electrophoresis. Part 1. Fundamentals. *Electroanalysis* **17**, 1198-1206 (2005).
3. Hutchinson, J.P. *et al.* Identification of inorganicions in post-blast explosive residues using portable CE instrumentation and capacitively coupled contactless conductivity detection. *Electrophoresis* **29**, 4593-4602 (2008).
4. Tanyanyiwa, J., Galliker, B., Schwarz, M.A. & Hauser, P.C. Improved capacitively coupled conductivity detector for capillary electrophoresis. *Analyst* **127**, 214-218 (2002).
5. Pumera, M. *et al.* Contactless conductivity detector for microchip capillary electrophoresis. *Anal. Chem.* **74**, 1968-1971 (2002).
6. Thredgold, L.D., Khodakov, D.A., Ellis, A.V. & Lenehan, C.E. On-chip capacitively coupled contactless conductivity detection using "injected" metal electrodes. *Analyst* **138**, 4275-4279 (2013).
7. Tanyanyiwa, J. *et al.* High-voltage contactless conductivity detection forl ab-on-chip devices using external electrodes on the holder. *Analys*t **128**, 1019-1022 (2003).
8. Brito-Neto, J.G.A., da Silva, J.A.F., Blanes, L. & do Lago, C.L. Understanding capacitively coupled contactless conductivity detection in capillary and microchip electrophoresis. Part 2. Peakshape, straycapacitance, noise, and actual electronics. *Electroanalysis* **17**, 1207-1214 (2005).
9. Collins, D.A., Nesterenko, E.P., Brabazon, D. & Paull, B. In-process phase growth measurement technique in the fabrication of monolithicporous layer open tubular (monoPLOT) columns using capacitively coupled contactless conductivity. *Analyst* **138**, 2540-2545 (2013).
10. Lee, T.M.-H., Carles, M.C. & Hsing, I.-M. Microfabricated PCR-electrochemical device for simultaneous DNA amplification and detection. *Lab Chip* **3**, 100-105 (2003).
11. Zhang, X. *et al*. Monitoring the progression of loop-mediated isothermal amplification (LAMP) using conductivity. *Anal. Biochem*. **466**, 16-18 (2014).
12. Notomi, T. *et al.* Loop-mediated isothermal amplification of DNA. *Nucleic Acids Res*. **28**, E63 (2000).
13. Tomita, N., Mori, Y., Kanda, H. & Notomi, T. Loop-mediated isothermal amplification (LAMP) of gene sequences and simple visual detection of products. *Nat. Protoc.* **3**, 877-882 (2008).
14. Veigas, B. *et al.* Ion sensing (EIS) real-time quantitative monitorization of isothermal DNA amplification. *Biosens Bioelectron*. **55**, 50-55 (2014).
15. Wang, F., Jiang, L., Yang, Q., Prinyawiwatkul, W. & Ge, B. Rapid and specific detection of *Escherichia coli* serogroups O26, O45, O103, O111, O121, O145, and O157 in ground beef, beef trim, and produce by loop-mediated isothermal amplification. *Appl. Environ. Microbiol.* **78**, 2727-2736 (2012).
16. Mori, Y., Nagamine, K., Tomita, N. & Notomi, T. Detection of loop-mediated isothermal amplification reaction by turbidity derived from magnesium pyrophosphate formation. *Biochem. Bioph. Res. Co.* **289**, 150-154 (2001).

17. Zhou, D. *et al*. Establishment and application of a loop-mediated isothermal amplification (LAMP) system for detection of cry1Ac transgenic sugarcane. *Sci. Rep-UK*. **4**, 4912 (2014).

18. Mori, Y. & Notomi, T. Loop-mediated isothermal amplification (LAMP): a rapid, accurate, and cost-effective diagnostic method for infectious diseases. *J. Infect. Chemother.* **15**, 62-69 (2009).

19. Goto, M., Honda, E., Ogura, A., Nomoto,A. & Hanaki, K. Colorimetric detection of loop-mediated isothermal amplification reaction by using hydroxynaphthol blue. *Biotechniques* **46**, 167-172 (2009).

20. Luo, L. *et al*. Visual detection of high-risk human papillomavirus genotypes 16, 18, 45, 52, and 58 by loop-mediated isothermal amplification with hydroxynaphtholblue dye. *J. Clin. Microbiol*. **49**, 3545-3550 (2011).

21. Safavieh, M. *et al*. A simple cassette as point-of-care diagnostic device for naked-eye colorimetric bacteria detection. *Analyst* **139**, 482-487(2014).

22. Salm, E. *et al*. Electrical detection of nucleic acid amplification using an on-chip quasi-reference electrode and a PVC REFET. *Anal. Chem.* **86**, 6968-6975 (2014).

23. Zhang, X., Jiao, K., Liu, S. & Hu, Y. A readily reusable electrochemical DNA hybridization biosensor based on the interaction of DNA with single-walled carbon nanotubes. *Anal. Chem.* **81**, 6006-6012 (2009).
